# Inhibition of CDK9 by voruciclib synergistically enhances cell death induced by the Bcl-2 selective inhibitor venetoclax in preclinical models of acute myeloid leukemia

**DOI:** 10.1038/s41392-020-0112-3

**Published:** 2020-02-26

**Authors:** Daniel A. Luedtke, Yongwei Su, Jun Ma, Xinyu Li, Steven A. Buck, Holly Edwards, Lisa Polin, Juiwanna Kushner, Sijana H. Dzinic, Kathryn White, Hai Lin, Jeffrey W. Taub, Yubin Ge

**Affiliations:** 1 Cancer Biology Graduate Program, Wayne State University School of Medicine, Detroit, MI 48201 USA; 2 School of Life Sciences, Jilin University, 130021 Changchun, China; 3 Division of Pediatric Hematology and Oncology, Children’s Hospital of Michigan, Detroit, MI USA 48201; 4 Department of Pediatrics, Wayne State University School of Medicine, Detroit, MI 48201 USA; 5 Department of Oncology, Wayne State University School of Medicine, Detroit, MI 48201 USA; 6 Molecular Therapeutics Program, Karmanos Cancer Institute, Wayne State University School of Medicine, Detroit, MI 48201 USA; 7 Department of Hematology and Oncology, The First Hospital of Jilin University, 130021 Changchun, China

**Keywords:** Haematological cancer, Drug development

## Abstract

Venetoclax, an FDA-approved Bcl-2 selective inhibitor for the treatment of chronic lymphocytic leukemia and acute myeloid leukemia (AML), is tolerated well in elderly patients with AML and has good overall response rates; however, resistance remains a concern. In this study, we show that targeting CDK9 with voruciclib in combination with venetoclax results in synergistic antileukemic activity against AML cell lines and primary patient samples. CDK9 inhibition enhances venetoclax activity through downregulation of Mcl-1 and c-Myc. However, downregulation of Mcl-1 is transient, which necessitates an intermittent treatment schedule to allow for repeated downregulation of Mcl-1. Accordingly, an every other day schedule of the CDK9 inhibitor is effective in vitro and in vivo in enhancing the efficacy of venetoclax. Our preclinical data provide a rationale for an intermittent drug administration schedule for the clinical evaluation of the combination treatment for AML.

## Introduction

Although the biology of acute myeloid leukemia (AML) has been well studied, the prognosis of this deadly disease remains frustratingly low, with 5-year survival rates of 66% in children and 24% in adults.^1^ The standard of care has changed little over the last several decades (cytarabine plus an anthracycline followed by bone marrow transplant), which has translated into only modest improvements in outcomes.^2^ The majority of AML patients are elderly and cannot tolerate intensive standard chemotherapy. For patients who are treated with intensive standard chemotherapy, relapse often occurs. Thus, better-tolerated and more effective therapies are needed to improve treatment outcomes of patients with AML.

The antiapoptotic Bcl-2 family proteins Bcl-2, Bcl-xL, and Mcl-1 are key apoptosis regulators, and their dysregulation causes resistance to chemotherapy and promotes survival of cancer cells.^3,4^ Bcl-2, Bcl-xL, and Mcl-1 have been found to be overexpressed in AML, and their overexpression is associated with resistance to chemotherapy and poor clinical outcomes.^5–10^ In particular, Bcl-2 is overexpressed in AML cells, making Bcl-2 a promising therapeutic target.^11^ Venetoclax (initially called ABT-199) is a BH3 mimetic and Bcl-2 selective inhibitor developed by AbbVie.^12,13^ Venetoclax improved upon prior Bcl-2 family inhibitors by not targeting Bcl-xL, whose inhibition leads to thrombocytopenia and clinical toxicity.^12^ Its excellent antileukemic activity against chronic lymphocytic leukemia (CLL) led to FDA approval of venetoclax in April 2016. For AML patients who cannot tolerate standard intensive chemotherapy, venetoclax in combination with azacitidine, decitabine, or low-dose cytarabine (ara-C) generated encouraging response rates and survival outcomes, which led to FDA approval in November 2018. While these early results are promising, the median overall survival was 14.2–15.2 months,^14,15^ leaving substantial room for improvement.

Antiapoptotic proteins, such as Bcl-2, function by sequestering proapoptotic proteins, such as Bim, preventing proapoptotic proteins from binding to proapoptotic activators Bax and/or Bak and inducing apoptosis. Venetoclax is able to disrupt the interaction between Bim and Bcl-2, freeing Bim to induce apoptosis.^16^ However, our lab and others have found that venetoclax treatment increases Mcl-1 protein level and its binding to Bim in venetoclax-resistant AML cell lines and primary patient samples, representing an intrinsic mechanism of resistance to venetoclax in AML.^3,17^ The small-molecule Mcl-1 inhibitor A-1210477 was able to sensitize AML cells to venetoclax by disrupting compensatory Bim-Mcl-1 binding,^18^ representing a promising direct approach to overcome intrinsic resistance to venetoclax in AML. Alternatively, downregulation of Mcl-1 might indirectly overcome this intrinsic mechanism of resistance to improve treatment of AML with venetoclax.

CDK9, a key protein in the positive transcription elongation factor complex b, is a known regulator of transiently expressed prosurvival genes, including but not limited to Mcl-1, Cyclin D1, and c-Myc.^19,20^ Inhibition of CDK9 with flavopiridol (alvocidib; in phase 2 clinical trials in AML and tolerated in elderly patients) can downregulate Mcl-1 and cooperate with venetoclax in AML.^21–23^ There, however, remain concerns about the off-target toxicity of flavopiridol. Thus, the second-generation CDK9 selective inhibitor voruciclib was developed.^24^ Voruciclib has greater specificity for CDK9 than flavopiridol, downregulates Mcl-1, and synergizes with venetoclax in diffuse large B cell lymphoma in vitro and in vivo. However, the molecular mechanisms underlying the synergistic interaction between venetoclax and CDK9 inhibition are not fully understood.

In this study, we found that voruciclib synergizes with venetoclax to induce apoptosis in both AML cell lines and primary patient samples. Further, voruciclib transiently downregulates Mcl-1, which plays a role in the synergy between voruciclib and venetoclax. Interestingly, downregulation of c-Myc by voruciclib also contributes to its synergy with venetoclax. Simultaneous inhibition of c-Myc and knockdown of Mcl-1 show further enhancement of apoptosis induced by venetoclax than inhibition of c-Myc or knockdown of Mcl-1 alone. Furthermore, an every other day schedule seems optimal for the combination of venetoclax and voruciclib in an AML cell line-derived xenograft model.

## Results

### Voruciclib inhibition induces apoptosis in AML cell lines and primary patient samples

**Fig. 1:**
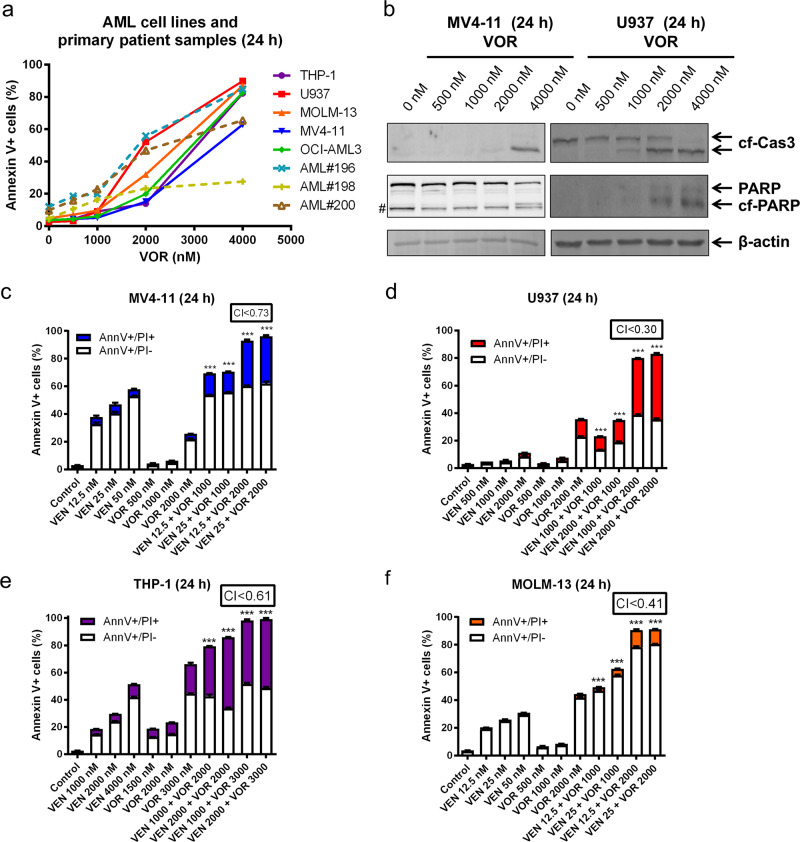
CDK9 inhibition synergizes with venetoclax to induce apoptosis in AML cells. **a** THP-1, U937, MOLM-13, MV4–11, and OCI-AML3 AML cell lines and primary patient samples were treated with voruciclib (VOR) for 24 h and then subjected to Annexin V-FITC/PI staining and flow cytometry analyses. **b** MV4–11 and U937 cells were treated with voruciclib for 24 h, and whole-cell lysates were subjected to western blotting and probed with the indicated antibodies. cf cleaved form; Cas3 caspase-3. # indicates a nonspecific band. **c**–**f** MV4–11, U937, THP-1, and MOLM-13 AML cell lines were treated with venetoclax (VEN) and voruciclib (VOR), alone or in combination, for 24 h and then subjected to Annexin V-FITC/PI staining and flow cytometry analyses. CI values were calculated using CompuSyn software. ***p < 0.001

To test the antileukemic activity of voruciclib, we tested various clinically achievable concentrations^25^ in five AML cell lines and three primary AML patient samples (Fig. 1a). Single drug treatment induced high levels of Annexin V+ cells (determined by Annexin V/PI staining and flow cytometry analyses) in all five AML cell lines and two of the three primary patient samples, with moderate activity in the third primary patient sample. Voruciclib treatment caused substantially increased cleavage of caspase-3 and PARP at higher concentrations (Fig. 1b), confirming the flow cytometry data, demonstrating that voruciclib induces apoptosis of AML cells. The low levels of cleaved caspase-3 and PARP detected for after treatment with 2 μM were likely due to the sensitivity of the assay.

### Voruciclib synergizes with venetoclax in AML cell lines

Having shown the antileukemic activity of single drug treatment, we sought to determine the effect of CDK9 inhibition on apoptosis induced by venetoclax. AML cell lines were treated with various concentrations of venetoclax and voruciclib, alone or in combination, for 24 h. The combination index (CI) was used to determine the extent and direction of antileukemic interactions.^26^ MV4–11, U937, THP-1, and MOLM-13 cell lines treated with venetoclax plus voruciclib at clinically achievable concentrations resulted in significantly increased levels of Annexin V+ cells compared to cell lines treated with a single drug (CI < 0.73; Fig. 1c–f), demonstrating the efficacy and synergy of the combination treatment.

### Transient Mcl-1 downregulation by voruciclib

**Fig. 2:**
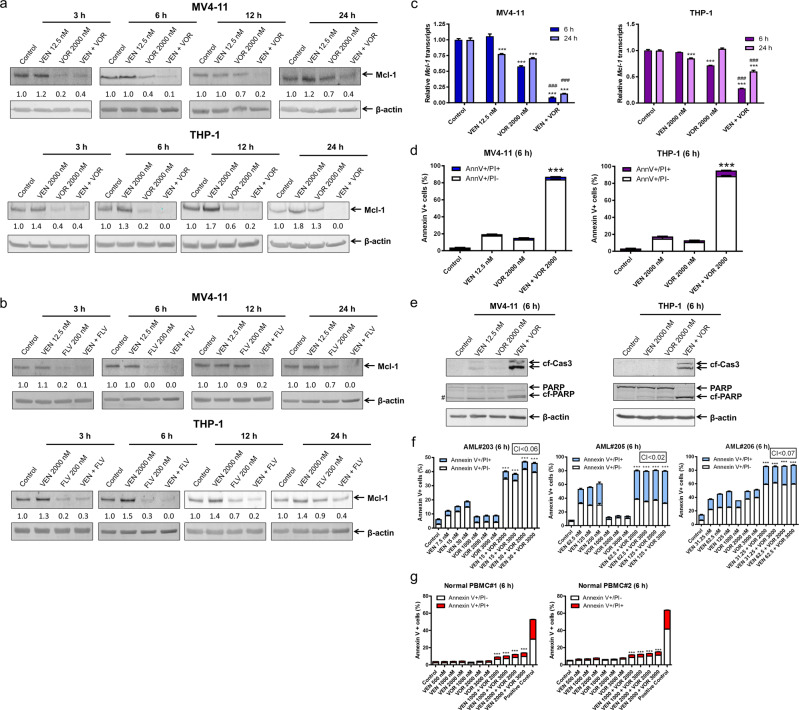
Transient Mcl-1 downregulation by voruciclib enhances apoptosis induced by venetoclax in AML cells. **a**, **b** THP-1 and MV4–11 cells were treated with venetoclax (VEN) and voruciclib (VOR) or flavopiridol (FLV), alone or in combination, for the indicated times. Whole-cell lysates were subjected to western blotting and probed with the indicated antibodies. Relative densitometry measurements were determined using Odyssey Software V3.0 and compared to the control and normalized to β-actin. **c** MV4–11 and THP-1 cells were treated with venetoclax or voruciclib, alone or combined, for 6 or 24 h. RNA was extracted with TRIzol and subjected to real-time RT‐PCR analysis and normalized to 18S rRNA. ***p < 0.001 compared to control, while ^###^*p* < 0.001 compared to individual drug treatment. **d**, **f**, **g** THP-1 and MV4–11 AML cell lines, primary AML patient samples, and normal peripheral blood mononuclear cells (PBMCs) were treated with venetoclax and voruciclib, alone or in combination, for 6 h and then subjected to Annexin V-FITC/PI staining and flow cytometry analyses. ***p < 0.001 compared to individual drug treatment. CI values were calculated using CompuSyn software. **e** MV4–11 and THP-1 cells were tr**e**ated with venetoclax and voruciclib, alone or in combination, for 6 h. Whole-cell lysates were subjected to western blotting and probed with the indicated antibodies. # indicates a nonspecific band

Next, we began to determine the mechanism by which the combination induced apoptosis, particularly focusing on modulation of Mcl-1. Consistent with previous studies by our lab,^17^ venetoclax-resistant cells (THP-1) but not venetoclax-sensitive cells (MV4–11) showed an increase in Mcl-1 protein in response to venetoclax treatment. Interestingly, CDK9 inhibition by voruciclib resulted in a transient downregulation of Mcl-1 protein, which rebounded 12 h after drug treatment in both MV4–11 and THP-1 cell lines (Fig. 2a). However, this transient downregulation by voruciclib was sufficient to maintain Mcl-1 levels below the control level when the cells were cotreated with venetoclax. Similar results were obtained using flavopiridol (Fig. 2b). Mcl-1 transcript levels decreased after voruciclib treatment alone and were further decreased by the addition of venetoclax, potentially due to apoptosis induced by the drug combination. Curiously, in THP-1, but not MV4–11, Mcl-1 transcripts rebounded in response to CDK9 inhibition, indicating that besides a transcriptional mechanism, the rebound of Mcl-1 protein may also involve modulation of protein stability (Fig. 2c).

Given the important role Mcl-1 plays in the intrinsic resistance to venetoclax in AML cells,^13,17,27^ we sought to determine whether transient downregulation of Mcl-1 would enhance venetoclax-induced apoptosis. At 6 h, voruciclib significantly and greatly enhanced the percent of annexin V+ cells induced by venetoclax (Fig. 2d). This was accompanied by greatly enhanced cleavage of caspase-3 and PARP by the combination treatments (Fig. 2e), demonstrating induction of apoptosis. To further increase the clinical relevance of our study, we tested three primary AML patient samples ex vivo and striking synergy was observed for venetoclax and voruciclib (CI < 0.07; Fig. 2f). Furthermore, the combination treatment had minimal effect on normal peripheral blood mononuclear cells (Fig. 2g). Taken together, our results show that voruciclib transiently downregulates Mcl-1, which may contribute to the synergy between voruciclib and venetoclax.

### The intrinsic apoptotic pathway is partially responsible for the cooperation of voruciclib and venetoclax to induce apoptosis

**Fig. 3:**
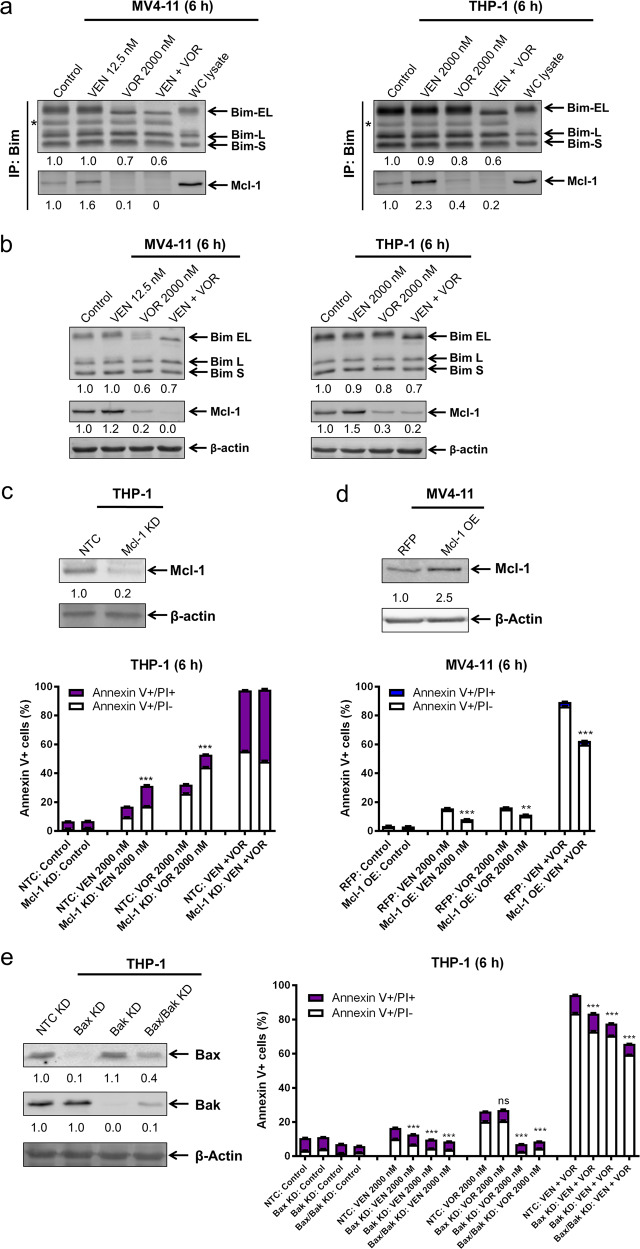
Apoptosis induced by voruciclib and venetoclax, alone or combined, is at least partially through the intrinsic apoptotic pathway. **a**, **b** MV4–11 and THP-1 cells were treated with venetoclax (VEN) and voruciclib (VOR) alone or in combination for 6 h. Bim was immunoprecipitated from whole-cell lysates and then subjected to western blotting and probed with the indicated antibodies. Relative densitometry measurements of Bim and Mcl-1 were measured using Odyssey Software V3.0 and normalized to the control. * indicates the light chain of IgG. Whole-cell (WC) lysate from the no drug treatment control was run on the same blot as the rest of the co-IP samples. Whole-cell lysates from the 6 h treatment with venetoclax alone or in combination with voruciclib are shown in panel **b**. **c**–**e** CRISPR knockdown (KD) of Mcl-1, pLOC overexpression (OE) of Mcl-1, and shRNA KD of Bax, Bak, and Bax/Bak were generated with the indicated control (non-target control [NTC] and red fluorescent protein [RFP]) in the indicated cell lines. Whole-cell lysates were subjected to western blotting and probed with the indicated antibodies to confirm the KD or OE. Relative densitometry measurements were determined using Odyssey Software V3.0 and compared to the control and normalized to β-actin. Cells were treated with venetoclax and voruciclib, alone or in combination, for 6 h and then subjected to Annexin V-FITC/PI staining and flow cytometry analyses. **p < 0.01, ***p < 0.001, and ns indicates not significant

To determine the role of Mcl-1 and the intrinsic apoptotic pathway in apoptosis induced by the combination, we performed co-immunoprecipitation of Bim and Mcl-1 in AML cell lines. CDK9 inhibition alone or in combination with venetoclax was able to greatly decrease the level of Mcl-1 protein bound to Bim in MV4–11 and THP-1 cells at 6 h (Figs. 3a and S1). It is important to note that there was a decrease in Bim protein levels in whole-cell lysates post voruciclib and its combination with venetoclax treatments (30–40% in MV4–11 cells and 20–40% in THP-1 cells; Fig. 3b). This decrease is reflected in the Bim immunoprecipitation results (Fig. 3a). These results suggest that the decrease in Bim bound to Mcl-1 was likely due to the greatly decreased Mcl-1 protein level in the cells (Fig. 3b). Nonetheless, voruciclib treatment, which decreases Bim bound to Mcl-1, in combination with venetoclax treatment, which decreases Bim bound to Bcl-2, should free Bim to induce apoptosis.

Mcl-1 knockdown (~80% compared to non-target control (NTC)) moderately but significantly enhanced single drug treatment but did not significantly affect the highly efficacious combination treatment (Fig. 3c). Conversely, Mcl-1 overexpression partially rescued the MV4–11 cells from single and combination treatment (Fig. 3d). To further test the role of the intrinsic apoptotic pathway, Bax, Bak, and Bax/Bak knockdown were performed. Knockdown of Bak, which has a higher affinity for Mcl-1 than Bax, completely rescued the cells from voruciclib treatment (Fig. 3e). Knockdown of Bax or Bak led to a modest rescue of the cells from venetoclax treatment and its combination with voruciclib. Bax/Bak double knockdown completely rescued the cells from voruciclib and venetoclax treatment while partially rescuing the cells from combination treatment (Fig. 3e).

### Downregulation of c-Myc and Mcl-1 enhances venetoclax-induced apoptosis

**Fig. 4:**
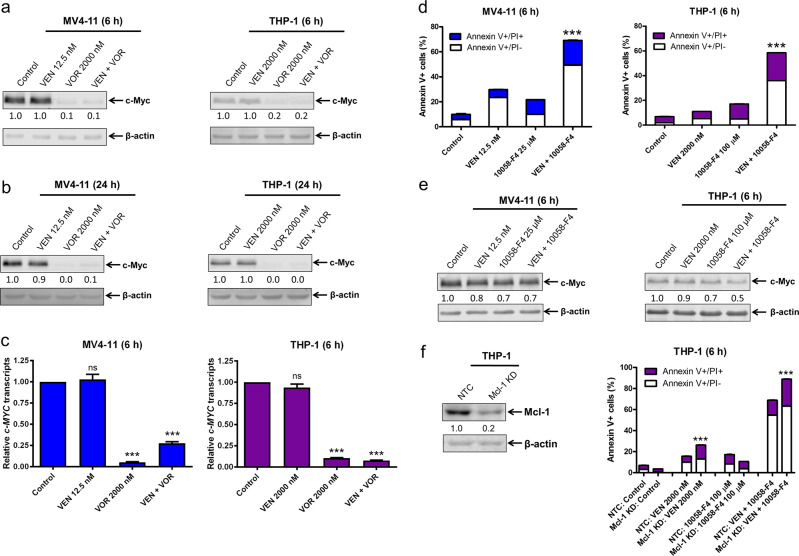
Downregulation of c-Myc and Mcl-1 enhances venetoclax-induced apoptosis. **a**, **b**, **e** MV4–11 and THP-1 cells were treated with venetoclax (VEN), voruciclib (VOR), or 10058-F4, alone or in combination for 6 or 24 h. Whole-cell lysates were subjected to western blotting and probed with the indicated antibodies. Relative densitometry measurements were determined using Odyssey Software V3.0, normalized to β-actin, and compared to the control. **c** RNA was extracted with TRIzol, subjected to real-time RT‐PCR analysis, and normalized to the 18S control. ***p < 0.001, ns indicates not significant. **d** MV4–11 and THP-1 cells were treated with venetoclax or 10058-F4, alone or in combination for 6 h and then subjected to Annexin V-FITC/PI staining and flow cytometry analyses. ***p < 0.001. **f** CRISPR knockdown (KD) of Mcl-1 was generated with the indicated control (NTC) in THP-1 cells. Whole-cell lysates were subjected to western blotting and probed with the indicated antibodies to confirm the KD. Cells were treated with ABT-199 and 10058-F4, alone or in combination, for 6 h and then subjected to Annexin V-FITC/PI staining and flow cytometry analyses. ***p < 0.001

CDK9 promotes transcription of genes encoding prosurvival factors, including c-Myc, which plays a critical role in leukemogenesis and drug resistance in AML.^22,28,29^ Thus, CDK9 inhibition may downregulate c-Myc, leading to enhancement of apoptosis induced by venetoclax. CDK9 inhibition for 6 h with voruciclib, alone or in combination with venetoclax, caused major downregulation of c-Myc protein levels in both MV4–11 and THP-1 cells (Fig. 4a). Unlike the effect on Mcl-1, c-Myc protein levels did not rebound after extended treatment with voruciclib (Fig. 4b). c-Myc transcript levels were also significantly decreased by voruciclib treatment alone and in combination with venetoclax (Fig. 4c), suggesting a transcriptional mechanism by which voruciclib downregulates c-Myc expression. To determine the functional contribution of c-Myc downregulation to the antileukemic activity of venetoclax, we used the c-Myc inhibitor 10058-F4. Treatment of MV4–11 and THP-1 cells with 10058-F4 modestly decreased c-Myc protein levels but significantly and substantially enhanced venetoclax-induced apoptosis (Fig. 4d, e), which was further enhanced when Mcl-1 was knocked down in these cells (Fig. 4f). These results demonstrate that both c-Myc and Mcl-1 are important factors involved in the synergy between venetoclax and voruciclib. Further, to achieve greater efficacy for the combination of voruciclib and venetoclax, both c-Myc and Mcl-1 need to be downregulated.

### Intermittent CDK9 inhibition enhances venetoclax activity in vitro and in vivo

**Fig. 5:**
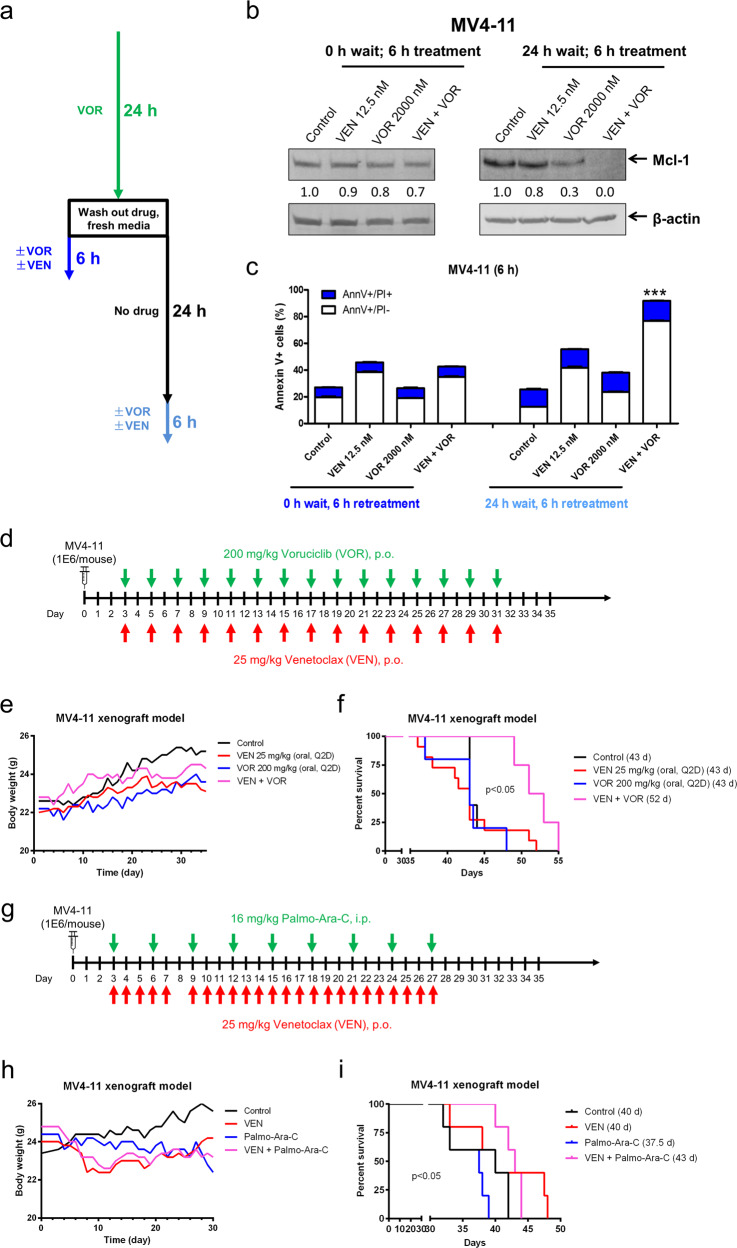
Intermittent CDK9 inhibition enhances venetoclax activity in vitro and in vivo. **a** Schematic for the rechallenge treatments. **b**, **c** MV4–11 cells were treated with voruciclib (VOR) for 24 h. The cells were spun and washed with PBS. Cells were cultured in drug-free medium for 0 or 24 h and then treated as indicated for 6 h. Whole-cell lysates from re-treated cells were subjected to western blotting and probed with the indicated antibodies. Relative densitometry measurements were determined using Odyssey Software V3.0 and compared to the control and normalized to β-actin. Re-treated cells were subjected to Annexin V-FITC/PI staining and flow cytometry analyses. ***p < 0.001. **d** In vivo treatment schema. NSGS mice were injected with 1 × 10^6^ MV4–11 cells via the tail vein and treated Q2D starting on day 3 with 25 mg/kg/inj ABT-199 p.o. and/or 200 mg/kg/inj voruciclib p.o. **e** Average mouse body weights for the treatment arms were measured on a daily basis. **f** Kaplan–Meier survival curves for the treatment arms are shown (Mantel–Cox statistical test). **g** In vivo treatment schema. NSGS mice were injected with 1 × 10^6^ MV4–11 cells via the tail vein and treated starting on day 3 with 25 mg/kg/inj ABT-199 p.o. daily and/or 16 mg/kg/inj palmo-ara-C i.p. Q3D. **h** Average mouse body weights for the treatment arms were measured on a daily basis. **i** Kaplan–Me**i**er survival curves for the treatment arms are shown (Mantel–Cox statistical test)

To determine whether re-treatment of AML cells with voruciclib will cause re-downregulation of Mcl-1, we designed experiments as shown in Fig. 5a. We started by treating MV4–11 cells for 24 h with 2000 nM voruciclib. Then, the cells were washed and resuspended in fresh media. The pretreated cells were treated with venetoclax and voruciclib alone or in combination for 6 h immediately after washing the cells or after a 24 h waiting period to allow the cells to “recover”. Re-treatment of the pretreated cells with voruciclib immediately after cell wash neither caused an obvious change in Mcl-1 protein levels nor significantly enhanced apoptosis induced by venetoclax (0 h wait, 6 h re-treatment; Fig. 5b, c). Conversely, pretreated cells that had a 24-h treatment “break” were more responsive to voruciclib alone and in combination with venetoclax based on the changes in Mcl-1 protein levels and the extent of apoptosis induction (24 h wait, 6 h re-treatment; Fig. 5b, c). These results were similar to those of flavopiridol (data not shown).

Based on our in vitro data of the CDK9 inhibitors, a Q2D dosing schedule was chosen for our in vivo studies. 1 × 10^6^ MV4–11 cells were injected into NSGS mice through the tail vein on day 0. Treatment started on day 3 and continued every other day until day 31 (the first day leukemia symptoms in the control mice were detected). A total of 15 injections were given for each single agent as monotherapy or in combination with mice receiving either 25 mg/kg/inj venetoclax p.o., 200 mg/kg/inj voruciclib p.o., or in combination, as shown in Fig. 5d. Body weight loss nadir for all treatment groups was ≤2.7%, indicative of well-tolerated treatment regimens (Fig. 5e). Venetoclax or voruciclib treatment did not extend median survival (43 days) compared to vehicle control treatment (43 days); however, the combination of voruciclib and venetoclax modestly improved median survival [52 days, *p* < 0.05, Mantel–Cox; 20.9% increase in lifespan (ILS); Fig. 5f]. We also tested a daily schedule using 130 mg/kg/inj voruciclib (p.o.; 6 days on and 1 day off) and 85 mg/kg/inj venetoclax in our MV4–11 mouse xenograft model (Fig. S2a). These doses were chosen because they were determined by a small toxicity trial to be the maximum tolerated dose for a daily schedule in this model (data not shown). The mice were treated for 2 weeks, given a weeklong drug holiday due to a 9.3% body weight loss in the combination treatment group, and then treated for another 2 weeks (Fig. S2b). ILS for venetoclax, voruciclib, and combination treated mice were 12%, 6.7%, and 14.7%, respectively (Fig. S2c). Next, we tested low-dose ara-C in combination with venetoclax, with venetoclax given on a daily basis and ara-C given every third day (Fig. 5g). Body weight loss nadir for all treatment groups was ≤ 8% (Fig. 5h). Median survival was 40 days for vehicle control and venetoclax-treated mice. Median survival of the ara-C-treated mice was 37.5 days, while combined venetoclax and ara-C treatment resulted in a median survival of 43 days (7.5% ILS; Fig. 5i). Taken together, our results demonstrate that voruciclib enhances the antileukemic activity of venetoclax in vivo and that the Q2D dosing schedule is effective both in vitro and in vivo.

## Discussion

In this study, we confirm the role of Mcl-1 downregulation by CDK9 inhibition in the enhancement of venetoclax activity in AML cells. Similar to our findings, Dey et al.^24^ reported downregulation of Mcl-1 in diffuse large B cell lymphoma cell lines after treatment with voruciclib for 6 h. In primary AML cells, Bogenberger et al.^21^ reported variable changes in Mcl-1 protein levels following flavopiridol treatment, similar to our results after 24 h treatment of the AML cell lines. They also observed synergistic antileukemic activity of combined venetoclax and flavopiridol in AML cells. Our results are in agreement and additionally show that voruciclib synergizes with venetoclax in AML cells. While the main target of voruciclib is CDK9, inhibition of CDK4 and CDK6 cannot be ruled out. However, Bogenburger et al.^21^ showed that selective inhibition of CDK4 and CDK6 does not potentiate venetoclax activity against AML cells. We also found that Bax/Bak knockdown partially rescued the cells from combination treatment (Fig. 3e). One potential explanation for this is the incomplete nature of the Bax and Bak knockdown; the remaining levels of Bax and Bak may be sufficient to induce apoptosis. Another potential explanation is the involvement of the extrinsic apoptotic pathway, as downregulation of c-Myc has been shown to increase expression of TRAIL.^30^

In addition to the confirmed hypothesized mechanism of Mcl-1 downregulation by CDK9 inhibition synergizing with Bcl-2 inhibition in AML, we found previously unrecognized novel mechanisms that will add to preclinical development as well as guide the use of CDK9 inhibitors in the clinic. While other studies have shown that Mcl-1 plays a role in the antileukemic activity of CDK9 inhibition in combination with venetoclax,^21,24,31^ we found that the CDK9 inhibitor-mediated downregulation of Mcl-1 was transient (Fig. 2a, b) and that continuous CDK9 inhibition no longer reduced Mcl-1 protein or enhanced venetoclax activity (Fig. 5c). However, an intermittent schedule was found to resume downregulation of Mcl-1 and enhance venetoclax activity. Further, a short intermittent administration schedule, e.g., every other day, resulted in a significant survival benefit for voruciclib in combination with venetoclax (Fig. 5f). Voruciclib in combination with venetoclax resulted in a 20.9% ILS, while low-dose ara-C in combination with venetoclax only increased the lifespan by 7.5%. MV4–11 cells are relatively resistant to ara-C in vitro (2 μM treatment for 24 h only results in 20% Annexin V-positive cells).^17^ When treated on a daily schedule, venetoclax, voruciclib, and the combination resulted in ILS of 12%, 6.7%, and 14.7%, respectively (Fig. S2b), supporting the use of an intermittent schedule, although further investigation in other AML xenograft mouse models is warranted. Thus, our data suggest that intermittent administration of voruciclib in combination with venetoclax may be a promising option for ara-C-resistant AML cases.

Another novel finding was the role of c-Myc in the synergistic activity of voruciclib in combination with venetoclax in AML. Mcl-1 overexpression only partially rescued the AML cells from venetoclax in combination with voruciclib treatment (Fig. 3d), indicating that downregulation of Mcl-1 was only part of the mechanism of action of the combination treatment. c-Myc inhibition significantly and greatly enhanced venetoclax activity (Fig. 4d). While knockdown of Mcl-1 further enhanced apoptosis induced by venetoclax in combination with c-Myc inhibition, the magnitude of enhancement was moderate. These results suggest that while downregulation of both c-Myc and Mcl-1 are important contributors to voruciclib’s enhancement of venetoclax activity in AML cells, c-Myc may be more important than Mcl-1. Further studies to determine how downregulation of c-Myc enhances venetoclax activity are needed but are beyond the scope of this manuscript.

In summary, voruciclib synergizes with venetoclax in AML cells. Voruciclib treatment results in downregulation of c-Myc and Mcl-1, which contribute to venetoclax activity. Voruciclib alone and in combination with venetoclax were observed to be more effective with an intermittent drug administration schedule in vitro and a Q2D schedule of voruciclib was effective in vivo. Based on our study, continuous combination treatment shows efficacy, although enhancement from downregulation of Mcl-1 is lost, suggesting that for optimal results, an intermittent schedule should be considered. Such a schedule may reduce toxicity, as the time between treatments will likely be prolonged in order to account for Mcl-1 rebound. In conclusion, our results support further clinical development of voruciclib in combination with venetoclax for the treatment of AML and provide evidence showing that CDK9 inhibitors should be administered with an intermittent schedule.

## Materials and methods

### Drugs

Venetoclax (ABT-199), flavopiridol (alvocidib), Z-VAD-FMK (a pan-caspase inhibitor), and 10058-F4 (a c-Myc inhibitor) were purchased from Selleck Chemicals (Houston, TX, USA). Voruciclib was provided by MEI Pharma (San Diego, CA, USA).

### Cell culture

The MV4–11, U937, and THP-1 cell lines were purchased from the American Type Culture Collection (2006, 2002, and 2014, respectively; Manassas, VA, USA). OCI-AML3 was purchased from the German Collection of Microorganisms and Cell Cultures (2011; DSMZ, Braunschweig, Germany). MOLM-13 was purchased from AddexBio (2012; San Diego, CA, USA). The cell lines were cultured in RPMI 1640 (except OCI-AML3, which was cultured in alpha-MEM) with 10–20% fetal bovine serum (Thermo Fisher Scientific, Waltham, MA, USA), 2 mM l-glutamine, 100 U/mL penicillin, and 100 μg/mL streptomycin. All cells were cultured in a 37 °C humidified atmosphere containing 5% CO_2_/95% air. The cell lines were authenticated in August 2017 at the Genomics Core at Karmanos Cancer Institute using the PowerPlex^®^ 16 System from Promega (Madison, WI, USA). Cell lines were tested for the presence of mycoplasma by PCR on a monthly basis.^32^

Diagnostic AML blast samples derived from patients were purified by standard Ficoll-Hypaque density centrifugation, and the isolated mononuclear cells were then cultured in RPMI 1640 with 20% fetal bovine serum, ITS solution (Sigma-Aldrich, St. Louis, MO, USA), and 20% supernatant of the 5637 bladder cancer cell line (as a source of granulocyte–macrophage colony-stimulating factor, granulocyte colony-stimulating factor, interleukin-1 beta, macrophage colony-stimulating factor, and stem cell factor^33–35^).

### Clinical samples

Diagnostic blast samples were obtained from the First Hospital of Jilin University. Written informed consent was provided according to the Declaration of Helsinki. This study was approved by the Human Ethics Committee of The First Hospital of Jilin University. Clinical samples were screened for FLT3-ITD, NPM1, C-kit, CEBPA, IDH1, IDH2, and DNMT3A gene mutations by PCR amplification and automated DNA sequencing and for fusion genes by real-time RT-PCR, as described previously.^33,36^ Patient characteristics are shown in Table 1.

**Table 1:** Patient characteristics of primary AML patient samples

Patients	Gender	Age (years)	Disease status	FAB subtype	Cytogenetics	Blast purity (%)	Gene mutation
AML#196	Female	40	Newly diagnosed	M5	46, XX	98.00	NPM1, FLT3-ITD, DNMT3A
AML#198	Female	44	Newly diagnosed	M2	46, XX, t(8;21)(q22;q22)	68.00	ASXL1, K-RAS, N-RAS
AML#200	Male	44	Newly diagnosed	M2	46, XY	77.00	CEBPAdm, FLT3-ITD
AML#203	Female	13	Newly diagnosed	M3	NA	NA	NA
AML#205	Female	61	Newly diagnosed	M3	46, XX, t(15;17): (q22;q21)	90.15	PML-RARα, ATRX, FLT3-ITD
AML#206	Male	36	Newly diagnosed	M1	46, XY	90.50	DNMT3A, IDH2, NPM1

*NA* not available

### Western blot analysis

Cells were lysed in the presence of protease and phosphatase inhibitors (Roche Diagnostics, Indianapolis, IN, USA). Whole-cell lysates were subjected to SDS-polyacrylamide gel electrophoresis, electrophoretically transferred onto polyvinylidene difluoride (PVDF) membranes (Thermo Fisher, Inc., Rockford, IL, USA), and immunoblotted with anti-Mcl-1 (4572), -PARP (9542), -Bim (2819), -Bak (3814), -Bax (2774), -c-Myc (5605s), -cleaved caspase-3 (9661, designated -cf-Cas3; Cell Signaling Technology, Danvers, MA, USA), or -β-actin (A2228; Sigma-Aldrich) antibody, as previously described.^37,38^ Immunoreactive proteins were visualized using the Odyssey Infrared Imaging System (Li-Cor, Lincoln, NE, USA), as described by the manufacturer. Western blots were repeated at least three times, and one representative blot is shown. Densitometry measurements were made using Odyssey V3.0 (Li-Cor), normalized to β-actin, and calculated as the fold change compared to the corresponding no drug treatment control.

### Annexin V-FITC/PI staining and flow cytometry analysis

AML cells were treated with venetoclax and voruciclib, alone or in combination, and subjected to flow cytometry analysis using the Annexin V-fluorescein isothiocyanate (FITC)/propidium iodide (PI) Apoptosis Kit (Beckman Coulter; Brea, CA), as previously described.^39,40^ Results are expressed as percent Annexin V-positive (Annexin V+) cells. For the AML cell lines, experiments were performed three independent times in triplicate, and the data presented are from one representative experiment, while the experiments with the patient samples were performed once in triplicate due to limited sample. Patient samples were chosen based on availability of adequate sample for the assay. The extent and direction of the antileukemic interaction was determined by calculating the combination index (CI) values using CompuSyn software (Combosyn, Inc., Paramus, NJ, USA). CI < 1, CI = 1, and CI > 1 indicate synergistic, additive, and antagonistic effects, respectively.^26,39^

### shRNA knockdown and pLOC overexpression

The pMD-VSV-G and delta 8.2 plasmids were gifts from Dr. Dong at Tulane University. Bax, Bak, and non-target control (NTC) shRNA lentiviral vectors were purchased from Sigma-Aldrich. Precision LentiORF Mcl‐1 and RFP (red fluorescent protein) lentivirus vectors were purchased from Dharmacon (Lafayette, CO, USA). Lentivirus production and transduction were carried out as previously described.^41^ Briefly, TLA-HEK293T cells were transfected with pMD-VSV-G, delta 8.2, and lentiviral shRNA or LentiORF constructs using Lipofectamine and Plus reagents (Thermo Fisher Scientific) according to the manufacturer’s instructions. Virus-containing culture medium was harvested 48 h post transfection. Cells were transduced overnight using 1 mL of virus supernatant and 4 μg of polybrene and then cultured for an additional 48 h prior to selection with puromycin or blasticidin.

### CRISPR knockdown

The lentiCRISPRv2 plasmid was a gift from Feng Zhang at the Broad Institute of MIT and Harvard (Addgene plasmid 52961). Guide RNAs were designed using the CRISPR design tool (http://crispr.mit.edu). The NTC (non-target control; 5′-GCACTACCAGAGCTAACTCA-3′) and Mcl-1 (5′-GCTTCCGCCAATCACCGCGC-3′) vectors were generated using Feng Zhang’s protocol, which is available on Addgene’s website (www.addgene.org). Lentivirus production and transduction were carried out as described above in “shRNA Knockdown,” but psPAX2 (a gift from Didier Trono at the Swiss Institute of Technology, Addgene plasmid #12260) was used instead of delta 8.2.

### Quantification of gene expression by real-time RT-PCR

Total RNA was extracted using TRIzol (Thermo Fisher Scientific), cDNAs were prepared from 2 µg of total RNA using random hexamer primers and an RT-PCR Kit (Thermo Fisher Scientific), and then purified using the QIAquick PCR Purification Kit (Qiagen, Valencia, CA, USA), as described previously.^40^ Mcl-1 mRNA (Hx01050896_m1) and 18s rRNA (Hs03928985_g1) were quantitated using TaqMan probes (Thermo Fisher Scientific) and a LightCycler 480 real-time PCR machine (Roche Diagnostics), based on the manufacturer’s instructions. The real-time PCR results are expressed as the mean from three independent experiments and were normalized to 18S transcripts. *c-Myc* transcripts were quantified using forward (5′-GTGGTCTTCCCCTACCCTCT-3′) and reverse (5′-CGAGGAGAGCAGAGAATCCG-3′) primers. These real-time PCR results are expressed as the mean from three independent experiments and were normalized to GAPDH transcripts measured by forward (5′-AGCCACATCGCTCAGACA-3′) and reverse (5′-GCCCAATACGACCAAATCC-3′) primers and SYBR Green. Fold changes were calculated using the comparative Ct method.^42^

### Cell line-derived xenografts

NSG-SGM3 mice (NSGS, JAX#013062; non-obese diabetic SCID gamma (NOD.Cg-Prkdc^scid^ Il2rg^tm1Wjl^ Tg(CMV-IL3, CSF2, KITLG)1Eav/MloySzJ; Jackson Laboratory, Bar Harbor ME, USA)) were injected intravenously (IV) with 1 × 10^6^ MV4–11 cells/mouse (day 0) and treated Q2D (every other day) from day 3 through day 31 (first day of leukemia symptoms detected in control mice) for a total of 15 injections. Treatment cohorts were administered 25 mg/kg/inj venetoclax p.o. and/or 200 mg/kg/inj voruciclib p.o. A group of vehicle-treated mice was included as a control, and five mice were included in all arms. The average mouse body weight for the study was 22.4 ± 1 g at the start of the treatment, with body weight and condition monitored 1–2 times daily for the duration of the study.

In a separate trial, NSGS mice were injected (IV) with 1 × 10^6^ MV4–11 cells/mouse (day 0) and treated from day 3 through day 27 (first day of leukemia symptoms detected in control mice). Treatment cohorts were administered 25 mg/kg/inj ABT-199 p.o. (daily for 5 days, one day off, followed by 19 days, for a total of 24 injections) and/or 16 mg/kg/inj Palmo-ara-C i.p. Q3D (every 3 days, for a total of 9 injections). A group of vehicle-treated mice was included as a control, and five mice were included in all arms. The average mouse body weight for the study was 24.2 ± 1 g at the start of the treatment, with body weight and condition monitored 1–2 times daily for the duration of the study.

The experimental endpoint and efficacy response for both trials were determined for each group based on the median day for development of leukemic symptoms (hindleg weakness, >15% weight loss, metastatic spread to internal organs). The %ILS was calculated: % ILS = [T-C/C] × 100, where “T” = treated and “C” = control median day of death. All mice were provided food and water ad libitum, given supportive fluids and supplements as needed, and housed within an AAALAC accredited animal facility with 24/7 veterinary care. All animal experiments were approved by the Institutional Animal Care and Use Committee of Wayne State University.

### Statistical analysis

Differences were compared utilizing a two-sample *t*-test. Error bars represent ± standard error of the mean (s.e.m.); significance level was set at *p* < 0.05 and adjusted for multiple comparisons with the Bonferroni correction. One-way ANOVA was used to compare differences between three or more groups with Dunnett correction when compared to the control or Bonferroni correction when compared to single drug treatments. Overall survival probability was estimated using the Kaplan–Meier method, and statistical analysis was performed using the Mantel–Cox test. All statistical analyses were performed utilizing GraphPad Prism 5.0.

## Supplementary information

Supplementary Figures S1 and S2

## Data Availability

The data supporting the findings of this study are available from the corresponding author upon reasonable request.
