# Supplementary material for: Inhibition of CDK9 by voruciclib synergistically enhances cell death induced by the Bcl-2 selective inhibitor venetoclax in preclinical models of acute myeloid leukemia
Source: Signal Transduct Target Ther. 2020 Feb 26;5:17. doi: 10.1038/s41392-020-0112-3 (PMC7042303; doi:10.1038/s41392-020-0112-3)
Supplement: Supplementary file 1 — Supplementary Figures S1 and S2 [file 41392_2020_112_MOESM1_ESM.docx]

Supplementary Materials for

Inhibition of CDK9 by Voruciclib Synergistically Enhances Cell Death Induced by the Bcl-2 Selective Inhibitor Venetoclax in Preclinical Models of Acute Myeloid Leukemia

Daniel A Luedtke, Yongwei Su, Jun Ma, Xinyu Li, Steven A. Buck, Holly Edwards, Lisa Polin5,6, Juiwanna Kushner, Sijana H Dzinic, Kathryn White, Hai Lin, Jeffrey W. Taub and Yubin Ge#

Correspondence to: gey@karmanos.org

**This PDF file includes:**

Figures S1 and S2


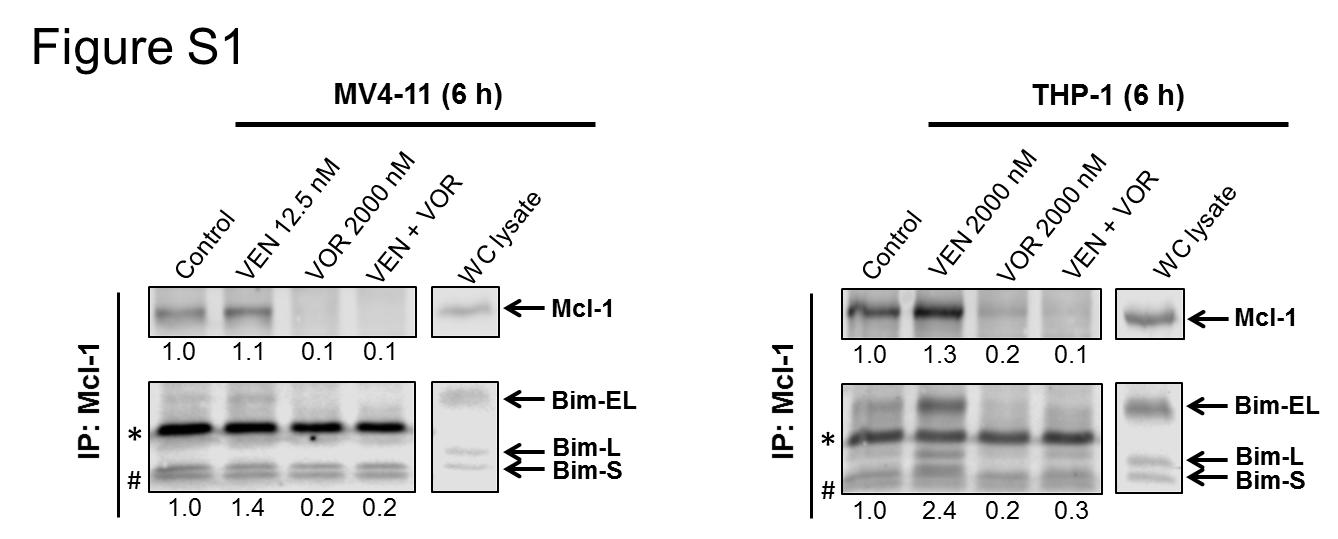


**Figure. S1.**

**Voruciclib decreases the level of Mcl-l protein bound to Bim.** MV4-11 and THP-1 cells were treated with venetoclax (VEN) and voruciclib (VOR) alone or in combination for 6 h. Mcl-1 was immunoprecipitated from whole cell lysates and then subjected to Western blotting and probed with the indicated antibodies. Relative densitometry measurements of Bim and Mcl-1 were measured using Odyssey Software V3.0 and normalized to the control. *indicates the light chain of IgG. # indicates a non-specific band (the lowest band shown). Whole cell (WC) lysate was run on the same blot as the rest of the samples; two unrelated lanes were cropped out.

**
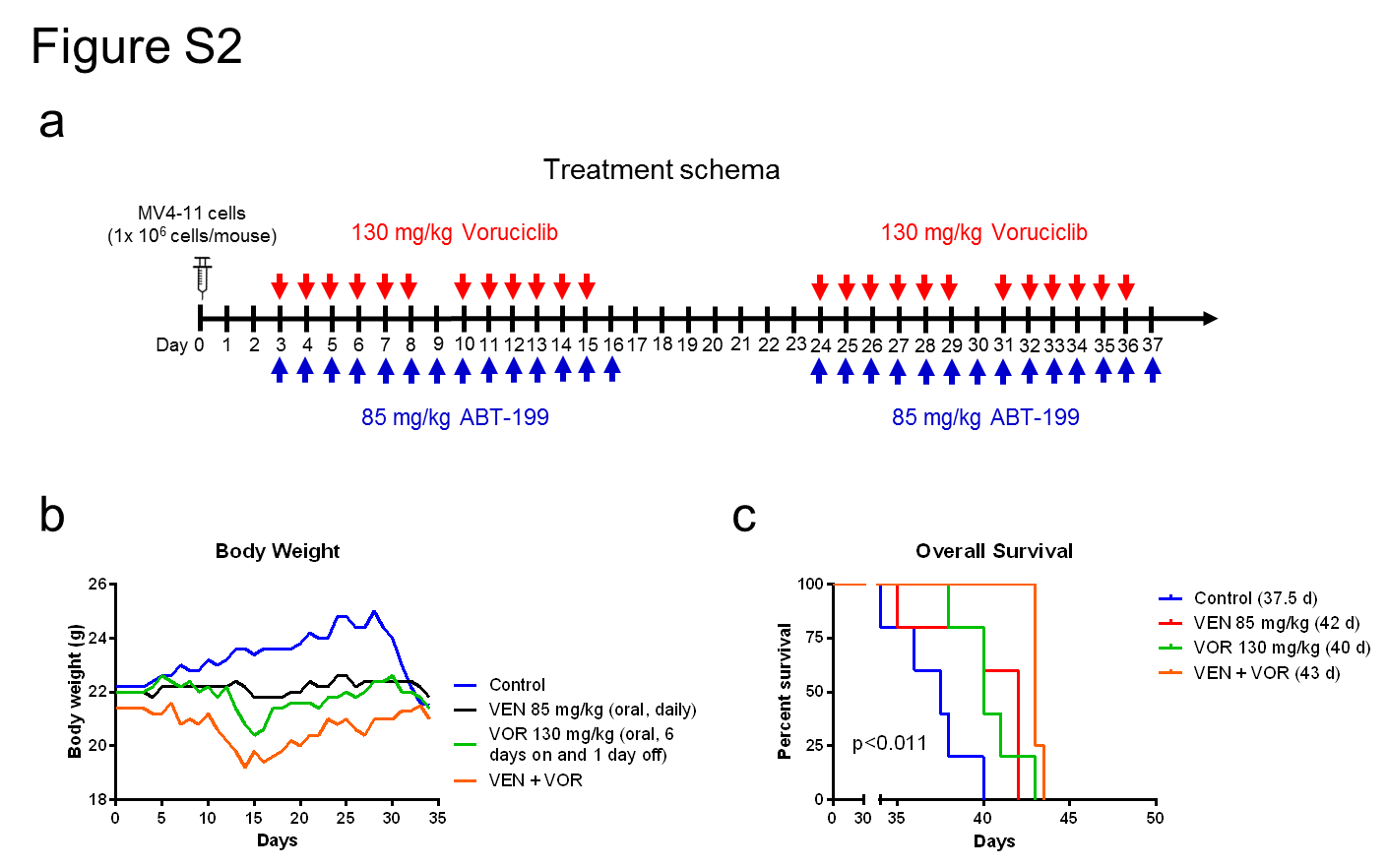
**

**Figure. S2.**

**CDK9 inhibition enhances venetoclax activity *in vivo*.** (**a**) *In vivo* treatment schema. NSGS mice were injected with 1x10^6^ MV4-11 cells via the tail vein and treated Q2D starting on day 3 with 85 mg/kg/inj ABT-199 p.o. and/or 130 mg/kg/inj voruciclib p.o.. (**b**) Average mouse body weights for the treatment arms were measured on a daily basis. (**c**) Kaplan-Meier survival curves for the treatment arms are shown (Mantel-Cox statistical test).
